# Why Are CD8 T Cell Epitopes of Human Influenza A Virus Conserved?

**DOI:** 10.1128/JVI.01534-18

**Published:** 2019-03-05

**Authors:** Zheng-Rong Tiger Li, Veronika I. Zarnitsyna, Anice C. Lowen, Daniel Weissman, Katia Koelle, Jacob E. Kohlmeier, Rustom Antia

**Affiliations:** a Department of Biology, Emory University, Atlanta, Georgia, USA; b Department of Microbiology and Immunology, Emory University School of Medicine, Atlanta, Georgia, USA; c Department of Physics, Emory University, Atlanta, Georgia, USA; Icahn School of Medicine at Mount Sinai

**Keywords:** influenza A virus, T cells, evolutionary biology, influenza vaccines, mathematical modeling

## Abstract

The high degree of conservation of CD8 T cell epitopes of influenza A virus (IAV) may allow for the development of T cell-inducing vaccines that provide protection across different strains and subtypes. This conservation is not fully explained by functional constraint, since an additional mutation(s) can compensate for the replicative fitness loss of IAV escape variants. Here, we propose three additional mechanisms that contribute to the conservation of CD8 T cell epitopes of IAV. First, influenza-specific CD8 T cells may protect predominantly against severe pathology rather than infection and may have only a modest effect on transmission. Second, polymorphism of the human major histocompatibility complex class I (MHC-I) gene restricts the advantage of an escape variant to only a small fraction of the human population who carry the relevant MHC-I alleles. Finally, infection with CD8 T cell escape variants may result in a compensatory increase in the responses to other epitopes of IAV. We use a combination of population genetics and epidemiological models to examine how the interplay between these mechanisms affects the rate of invasion of IAV escape variants. We conclude that for a wide range of biologically reasonable parameters, the invasion of an escape variant virus will be slow, with a timescale of a decade or more. The results suggest T cell-inducing vaccines do not engender the rapid evolution of IAV. Finally, we identify key parameters whose measurement will allow for more accurate quantification of the long-term effectiveness and impact of universal T cell-inducing influenza vaccines.

**IMPORTANCE** Universal influenza vaccines against the conserved epitopes of influenza A virus have been proposed to minimize the burden of seasonal outbreaks and prepare for the pandemics. However, it is not clear how rapidly T cell-inducing vaccines will select for viruses that escape these T cell responses. Our mathematical models explore the factors that contribute to the conservation of CD8 T cell epitopes and how rapidly the virus will evolve in response to T cell-inducing vaccines. We identify the key biological parameters to be measured and questions that need to be addressed in future studies.

## Abstract (precis)

Universal influenza vaccines against the conserved epitopes of influenza A virus have been proposed to minimize the burden of seasonal outbreaks and prepare for the pandemics. However, it is not clear how rapidly T cell-inducing vaccines will select for viruses that escape these T cell responses. Our mathematical models explore the factors that contribute to the conservation of CD8 T cell epitopes and how rapidly the virus will evolve in response to T cell-inducing vaccines. We identify the key biological parameters to be measured and questions that need to be addressed in future studies.

## INTRODUCTION

Seasonal influenza is a major public health concern, causing about 410,000 deaths worldwide annually (1). The inactivated vaccine currently in use requires an antigenic match between the vaccine and circulating strains. Antibodies induced by vaccination or exposure to earlier influenza A virus (IAV) strains drive the selection of antibody-escaping mutants (2) and result in the rapid evolution of antibody epitopes on hemagglutinin (HA) and neuraminidase (NA). These new drifted strains of the virus are responsible for seasonal outbreaks of influenza. In addition to the gradual changes caused by antigenic drift, larger antigenic shifts may occur when new antigenic subtypes (e.g., H1, H3, H5, and H7) emerge from zoonotic reservoirs into the human population. Both antigenic drift and antigenic shift necessitate frequent updating of the influenza vaccine. New approaches that focus on antibodies against the conserved regions of HA or CD8 T cells specific to the conserved epitopes on interior proteins have been proposed for the development of vaccines that have broad efficacy and ideally confer universal protection against all IAV subtypes (3–5). An important question, relevant to the development of a universal T cell-inducing vaccine, is whether IAV is likely to evolve and escape from the vaccine-induced CD8 T cell immunity. In this paper, we address this question by identifying the factors that are responsible for the conservation of the CD8 T cell epitopes of IAV. This allows us to quantify how rapidly the virus might evolve to escape from CD8 T cell responses generated by a universal T cell-inducing influenza vaccine.

CD8 T cells detect and kill virus-infected cells by recognizing short viral protein-derived peptides (epitopes) bound to major histocompatibility complex class I proteins (MHC-I) on the cell surface. While CD8 T cells do not generate sterile immunity that prevents infection, they can reduce the severity of disease and potentially viral transmission (6, 7). In addition, they may provide broad protection, because CD8 T cell epitopes of influenza A virus are largely conserved among drifted strains within one subtype and across different subtypes (7–9). The conservation of CD8 T cell epitopes is consistent with the observation that internal viral proteins (i.e., the nucleoprotein [NP] and matrix-1 protein [M1]), which harbor the majority of CD8 T cell epitopes, have a much lower substitution rate than HA and NA, which are the targets of antibody responses (10). Unlike the highly variable antibody epitopes, to date only 6 out of 64 CD8 T cell epitopes in human IAV have been found to have mutations that allow escape from CD8 T cell responses (9, 11).

Why are CD8 T cell epitopes of human influenza A virus conserved? The functional constraint hypothesis proposes that CD8 T cell epitopes are on the protein regions that do not tolerate changes in the amino acid sequence (12, 13). In this hypothesis, nonsynonymous mutations in the epitope region totally or partially diminish protein function and result in viruses that incur a substantial cost at the level of replication. In support of this, several studies have revealed that a single amino acid change at some residues within the NP or M1 can reduce the replication of the virus (12). A more recent study has shown that epistatic interactions may stabilize mutations and permit previously inaccessible destabilizing mutations (14). While the epistatic interactions greatly ameliorate the fitness cost, it would slow the rate at which a competitive mutant is generated. In addition, a number of other factors may contribute to the conservation of CD8 T cell epitopes. First, while CD8 T cells protect the hosts from severe pathology, they might not prevent infection and may only provide a modest reduction in virus transmission from an infected individual to new hosts. Second, there are multiple CD8 T cell epitopes, so an escape variant at one of these epitopes will still be targeted by T cells against the other epitopes. Third, a mutant that has a mutated CD8 T cell epitope will have a selective advantage only in the hosts whose MHC-I present the wild-type form of the CD8 T cell epitope; thus, polymorphism of MHC-I genes limits the selective advantage of a mutant to a fraction of the host population. In this paper, we use simple mathematical models to explore how the interplay between the above-mentioned factors affects the rate of invasion of a mutant, which has a mutated epitope that is not detected by CD8 T cells specific for the wild-type epitope. For brevity, the two virus populations are denoted wild-type, WT, and escape variant or mutant, MT.

(This article was submitted to an online preprint archive [15].)

## RESULTS

As mentioned in the Introduction, there are (at least) three factors that can affect the fitness of an escape variant: (i) the fitness cost of the mutation, (ii) the extent of selection for the escape variant in hosts carrying the relevant MHC-I allele(s) (i.e., one that presents the wild-type form of focal epitope), and (iii) the frequency of hosts with the relevant MHC-I allele(s) in the population. We begin with a relatively simple population genetics model that allows us to examine how the interplay between these factors affects the rate of invasion of an escape variant. This model assumes the selective advantage of the escape variant in a host is fixed. We then consider why this assumption may not hold and examine the consequences of relaxing this assumption, using an epidemiological model for the spread of the wild type and escape variant.

### Population genetics model.

Let us consider the wild type (WT) and one escape variant (MT) that carries a mutated focal epitope (Table 1). Let *h* represent the set of MHC-I allele(s) that presents the focal epitope of WT but not MT, and let *f* equal the frequency of *h*. The MHC-I alleles that present epitopes other than the focal one, *H*, are at frequency (1 – *f*). With the usual assumptions for Hardy-Weinberg equilibrium, we have the values shown. *m*, *s*, and *r* denote the fitness cost of MT in all hosts, the selective advantage of MT in hosts of *hh* genotype, and the dominance coefficient of the *h* allele, respectively. Here, we have assumed that the WT is equally fit in all genotypes, i.e., while *h* alleles present the focal epitope, the *H* alleles present other epitopes carried by the virus, so that all alleles confer about the same level of resistance. This is not true for the MT: while the *H* alleles still successfully present their other epitopes, *h* does not present its focal epitope. The frequency of the MT in generation *t*, *q_t_*, is given by

(1) qt=qt−1K1−qt−1+qt−1K

where *K* = (1 – *f*)^2^(1 – *m*) + 2*f*(1 – *f*)(1 – *m* + *rs*) + *f*^2^(1 – *m* + *s*) is the mean fitness of MT in the host population.

**TABLE 1:** Population genetics model

Parameter	Value for host genotype:		
	*HH*	*Hh*	*hh*
Genotype frequency	(1 − *f*)^2^	2*f*(1 − *f*)	*f*^2^
Relative fitness of WT	1	1	1
Relative fitness of MT	1 − *m*	1 − *m* + *rs*	1 − *m* + *s*

Equation 1 allows us to examine how the rate of invasion of the MT depends on *m*, the fitness cost; *f*, the frequency of *h*; *s*, the selective advantage MT accrued in the hosts of genotype *hh*; and *r*, the dominance coefficient as the ratio of selective advantage of MT in *Hh* hosts to that in *hh* hosts. The dominance coefficient depends on a number of factors, such as (i) the immunodominance of the focal epitope and (ii) the relationship between the magnitude of CD8 T cell responses and the rate of clearance of infected cells. In the absence of a quantitative understanding of these factors, we set *r* at an intermediate value (*r* = 0.5), and in Appendix 1 we discuss the consequences of changing *r* to lower or higher values.

**FIG 1:**
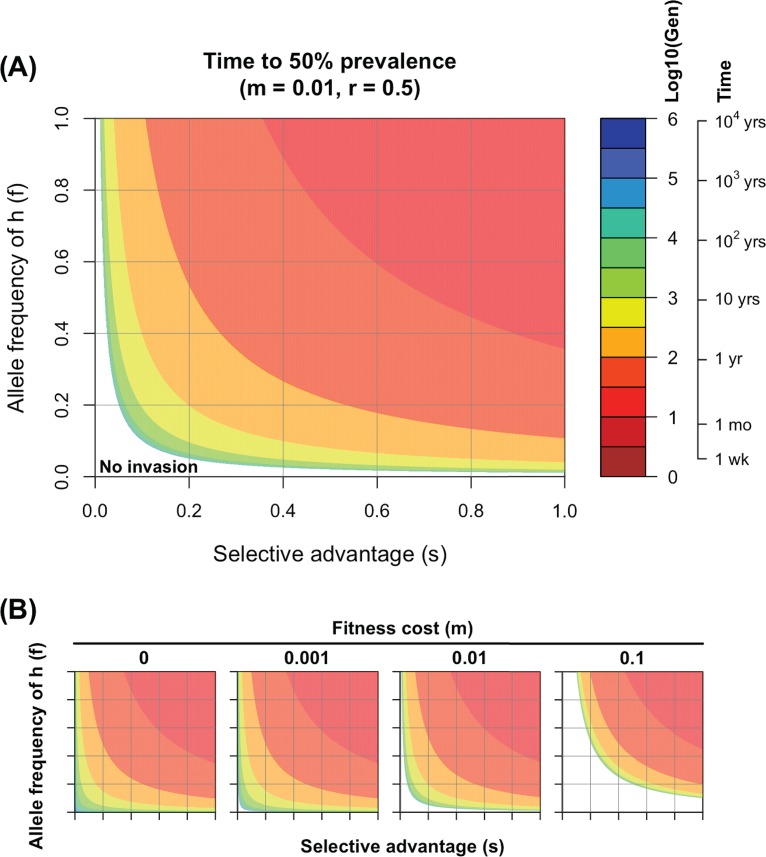
Rate of invasion of a CD8 T cell escape variant. (A) Contour plots show the number of generations on a log scale required for the escape variant to increase from 0.01% to 50% prevalence, predicted by equation 1. The approximate time for this to occur is calculated by assuming a serial interval of 3 to 4 days between infections (i.e., there are about 100 generations per year). Fitness cost is set to 1% (i.e., *m* = 0.01). (B) Time for invasion under different fitness costs, which goes from 0% to 10%. Even when the mutant does not have a fitness cost (*m* = 0), it will invade relatively slowly if *s* and *f* are small due to the nature of CD8 T cell protection and extent of MHC polymorphism. Ticks on the axes in panel B indicate the same numbers as those in panel A.

In Fig. 1, we plot how the rate of invasion depends on the selective advantage, *s*, of the MT (*x* axis) and the frequency, *f*, of the MHC-I alleles in which MT has a selective advantage (*y* axis). The rate of invasion is plotted on a log scale showing the log_10_ number of generations required for the MT to go from a prevalence of 0.01% to 50% in the host population. Given that the serial interval for influenza is about 3 to 4 days (16), 100 generations corresponds to about 1 year. In Fig. 1A, we set the fitness cost to 1% (i.e., *m* = 0.01). There is a parameter regime (white region with low *s* and *f*) where the fitness cost is sufficient to prevent MT from invasion. When MT can invade, we see faster invasion when *s* or *f* increases.

### Parameter *m*.

We show the effect of changing the fitness cost of MT (*m*) from 0% to 10% in Fig. 1B. We see that even if the escape variant does not have any fitness cost (i.e., *m* = 0), it invades relatively slowly when *s* and *f* are small (the region in the bottom left of the leftmost panel of Fig. 1B). See Appendix 1 for more details.

### Parameter *s*.

Building on the earlier ideas proposed by Halloran et al. (17), immunity can provide protection by reducing susceptibility of immune individuals to infection (IE*_S_*), as well as by reducing pathology (IE*_P_*) and transmission (IE*_I_*) in infected individuals. While the role of CD8 T cells in providing protection against influenza remains to be fully understood, a number of studies suggest that they play a significant role in reducing pathology (high IE*_P_*). In humans, a greater number of IAV-specific CD8 T cells prior to heterosubtypic virus infection is associated with faster viral clearance (6) and fewer symptoms (7). In support of human studies, mouse experiments have shown the cellular immunity induced by H1N1 and/or H3N2 is able to protect the hosts from lethal infection with avian H5N1 or H7N9 (18–20). CD8 T cells are likely to be less effective in preventing infection (very low IE*_S_*), although they may reduce the viral load during infection (modest IE*_I_*). Consequently, the selection pressure on a virus imposed by all CD8 T cell responses, 1 − (1 − IE*_S_*) (1 − IE*_I_*), will be relatively low. Furthermore, since a virus has multiple CD8 T cell epitopes, the selective advantage of a variant that escapes CD8 T cell responses to a single epitope would be considerably smaller (see Appendix 2 for details).

In conclusion, although CD8 T cells may provide some protection against severe pathology, escape variants having a mutated CD8 T cell epitope are unlikely to have much selective advantage, even in the hosts with the relevant MHC-I that presents the wild-type epitope. In other words, we expect *s* to be small.

### Parameter *f*.

**FIG 2:**
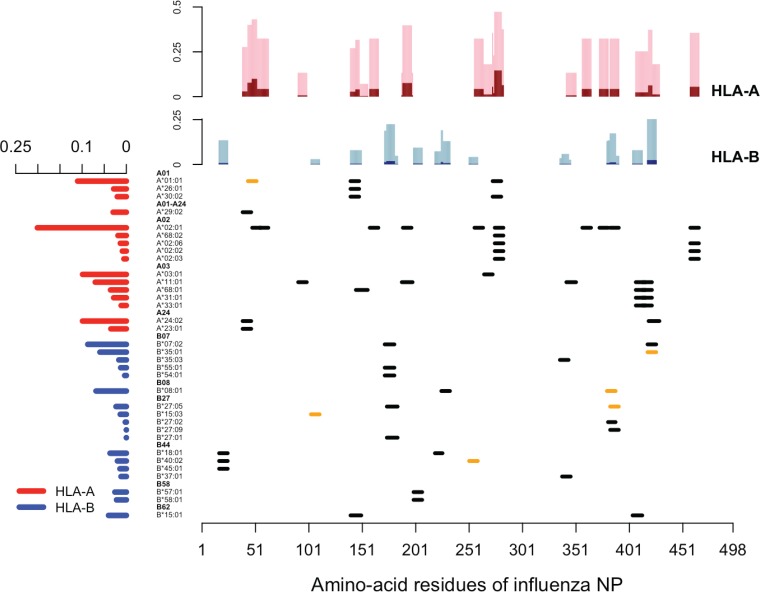
Distribution of CD8 T cell epitopes derived from the nucleoprotein of human IAV. In the main panel, each segment represents one unique CD8 T cell epitope derived from nucleoprotein of human IAV, aligned with its relevant HLA allele. Epitopes that have empirically verified escape mutants reported are labeled in orange. The bar graph on the left shows the weighted average frequency of the alleles, which are grouped into 11 supertypes (highlighted in boldface). The bar graph at the top shows the fractions of the population in which the virus with a mutation in the corresponding amino acid may have a selective advantage. The fractions of homozygote are shown in dark shades (dark red for HLA-A and dark blue for HLA-B), and the fractions of heterozygote are in light shades (pink for HLA-A and light blue for HLA-B).

Escape variants will accrue advantages in *hh* hosts and, to a lesser extent (proportional to the dominance coefficient, *r*), in *Hh* hosts. For example, the R384G escape mutation on the NP_383–391_ epitope is advantageous in the hosts who carry B*08:01 or B*27:05 but not in those who do not carry these alleles. In Fig. 2, we show the distribution of experimentally verified CD8 T cell epitopes derived from the nucleoprotein of human IAV retrieved from the Immune Epitope Database. It is clear that not a single epitope is presented by all human leukocyte antigen (HLA) alleles, and there is no single HLA allele presenting all epitopes. With this information and the frequencies of HLA alleles based on the National Marrow Donor Program (NMDP) data set, we estimated the fraction of host population where the mutation at each amino acid residue would confer a selective advantage (Fig. 2, top). We see that mutations typically confer selective advantage to the virus in only a small fraction of the human population.

### Integrating parameters *s*, *f*, and *m*.

As mentioned in the Introduction, epistatic interactions allow the virus to potentially generate escape variants to a given CD8 T cell epitope without engendering a substantial fitness cost. Our results show that since *s* and *f* are small, these escape variants will spread very slowly in the host population. For example, when *f* = 0.1 (i.e., the escape variant has advantages in about 20% of the infections, who are of *hh* genotype [*f*^2^ = 0.01] and of *Hh* genotype [2*f*(1 – *f*) = 0.18]), an escape variant would spend 10 years reaching 50% prevalence, even if its fitness is 10% more than that of the wild type (i.e., *s* = 0.1) in the hosts with relevant HLA, and no fitness cost is included (i.e., *m* = 0).

In conclusion, even if mutations that allow the virus to escape CD8 T cells specific for a given epitope have little or no fitness cost, escape variants will increase in frequency very slowly.

### Epidemiological models. (i) Compensatory immunity reduces the selective advantage of mutants over time.

The population genetics framework described above assumes that the fitness of an escape variant virus depends on host genotype and does not change over time. In particular, we assume that the fitness of escape variant (MT) relative to the WT equals (1 − *m* + *s*), (1 – *m* + *rs*), and (1 – *m*) in the hosts of *hh*, *Hh*, and *HH* genotypes, respectively. However, in a host of *hh* or *Hh* genotype who has been infected by MT, recovered, and moved to the susceptible category (due to antigenic drift), we might expect the selective advantage (*s*) of MT to decrease. This decline in *s* could arise for at least two biological reasons. First, the mutated epitope is still presented by the MHC-I; the mutation simply changes the configuration of the epitope recognized by the CD8 T cell receptor (12). In this case, the mutant epitope may induce a new set of CD8 T cells. Second, the absence of CD8 T cell response to one epitope could result in compensatory increases in responses to other epitopes.

**FIG 3:**
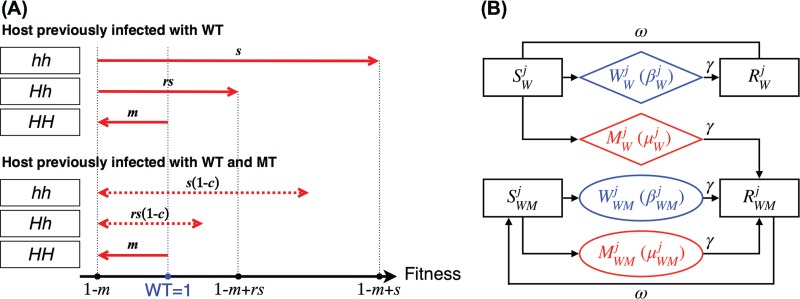
(A) Compensatory immunity alters the fitness of escape variant infection in the *Hh* and *hh* individuals. We show the viral fitness in the hosts of different genotypes and infection histories (being previously infected by the WT only or both WT and MT). The fitness of WT infection is 1 in all hosts (shown as the blue dot on the fitness scale). The fitness of MT in *HH*, *Hh*, and *hh* hosts are (1 – *m*), (1 – *m* + *rs*), and (1 – *m* + *s*), respectively (shown as the red arrows in the top panel and black dots on the fitness scale). Subsequent MT infections of *Hh* and *hh* hosts result in lower viral fitness, [1 – *m* + *rs*(1 – *c*)] and [1 – *m* + *s*(1 – *c*)], which is dependent on the extent of compensatory immunity and reduces the selective advantage by *c* and the dominance coefficient, *r* (shown as the red dashed arrows in the bottom panel). (B) Diagram illustrating the epidemiology of infections with WT (*W*, shown in blue) and MT (*M*, shown in red) in any of the three genotypes. Susceptible and immune hosts are indicated by *S* and *R*, respectively. The host genotype is indicated by the superscript *j* (*j* = 1, 2, and 3 for *HH*, *Hh*, and *hh*), and the prior infection status is indicated by a subscript (*W* for WT and *WM* for WT and MT).

We show the fitness of WT or MT infections in the hosts of different genotypes in Fig. 3. In hosts of *HH*, the fitness of WT and MT are 1 and (1 – *m*), the same as those in the population genetics model. The fitness of MT in hosts of *Hh* and *hh* that are infected with MT for the first time are (1 – *m* + *rs*) and (1 − *m* + *s*), as described earlier. After the hosts of *Hh* and *hh* recover and regain susceptibility due to antigenic drift, the fitness of MT following reinfection with MT becomes [1 – *m* + *rs*(1 – *c*)] and [1 – *m* + *s*(1 – *c*)], where *c* denotes the extent of compensatory CD8 T cell responses. Parameter *c* ranges from 0 to 1, where 0 corresponds to no compensatory increase in responses to other epitopes and 1 corresponds to full compensation. The ranges for the fitness of MT in hosts of *Hh* and *hh* with prior WT and MT infections are shown by the dashed arrows in Fig. 3A.

### (ii) Epidemiology of infections with wild-type and escape variant viruses.

We used a simple epidemiological model to describe the changes in frequencies of susceptible (*S*), infected (*W* and *M* for WT and MT infections), and immune (*R*) hosts. The subscript to *S*, *W*, *M*, and *R* populations indicates the viruses these hosts have been exposed to in the past. Individuals can be infected multiple times during their lifetime due to antigenic drift at antibody epitopes (21). We incorporate this by letting individuals move from the immune (*R*) to susceptible (*S*) compartments at rate ω (22).

We considered the epidemiology of WT infections in individuals of different genotypes, all of which have the same structure as that shown in Fig. 3B. Prior to the introduction of MT, we assumed that the WT is circulating and hosts have CD8 T cell immunity to the wild-type epitope. Due to antigenic drift, individuals typically get reinfected with a drifted strain every 5 to 10 years (21), and we chose the rate of loss of immunity corresponding to this duration (ω = 5 × 10^−4^/day, approximately once per 5.5 years). We began the simulations with WT infections at equilibrium.

On the introduction of MT, the MT has fitness (1 – *m*), (1 – *m* + rs), or (1 − *m* + s) in the MT-infected hosts of the *HH*, *Hh*, or *hh* genotype. MT-infected individuals move to the immune category with a subscript of WM (i.e., *R*_WM_). Individuals in *R*_WM_ become susceptible due to antigenic drift in the virus and move to *S*_WM_. When individuals in *S*_WM_ are infected with the WT, they move to *W*_WM_ and the WT has fitness of 1; however, when individuals in *S*_WM_ are infected with the MT, they move to *M*_WM_ and the MT has fitness (1 – *m*), [1 – *m* + rs(1 – c)], and [1 – *m* + rs(1 – c)] according to the host's genotype. For simplicity, we incorporate the fitness in the transmissibility parameter, β.

**FIG 4:**
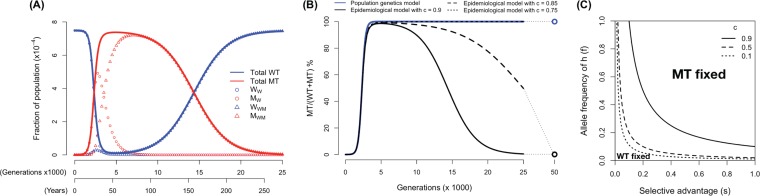
(A) The frequency of WT and escape variant (MT) infections as a function of time. When CD8 T cell responses are compensated for and, thus, decrease the selective advantage, the MT only invades transiently and goes extinct in the long run (*m* = 0.001, *s* = 0.05, *f* = 0.1, *r* = 0.5, and *c* = 0.9). (B) MT prevalence predicted by the epidemiological model with different extents of compensation (black lines) compared to the population genetics model (blue line). In all scenarios, the initial invasions of the MT (before it reaches 50% prevalence) are similar. After it reaches 50%, if the extent of compensation is smaller than the threshold (in our parameter setting, c* = 0.8; see equation 2), and then MT goes to fixation only slightly slower than that predicted by the population genetics model. In contrast, if the extent of compensation is higher than the threshold, the MT invades transiently and becomes extinct in the long run. (C) The parameter regimes of *s* and *f* where either MT or WT becomes fixed (and the other becomes extinct). We see that as the extent of compensation rises, the parameter regime where MT becomes fixed shrinks.

In Fig. 4, we explore how the escape variant (MT) spreads through the host population following its introduction. In particular, we focus on how compensatory immunity changes the outcomes predicted by the population genetics models. In Fig. 4A, we chose a simple scenario where the MT has a very small fitness cost (*m* = 0.001), 5% and 2.5% selective advantage (*s* = 0.05, *rs* = 0.025) in the hosts of *hh* and *Hh* genotypes, which accounts for 1% and 18% of the population (*f* = 0.18), respectively, and compensatory immunity reduces the selective advantage by 90% (*c* = 0.9). In this scenario, we see that the MT now only transiently invades, and compensation in host immunity causes the frequency of MT to decline as the population-level immunity against the MT increases. In Fig. 4B, we explore the consequences of changing the extent of compensation (*c*). We see that the initial rate of invasion is very similar to what is predicted by the population genetics model. However, once the MT has spread through the population, the outcome depends strongly on the extent of compensatory immunity described by the parameter *c*. If *c* is small, then the MT goes to fixation in a manner similar to that of the population genetics model. If *c* exceeds a threshold value, c*, given by

(2) c*=1−msf2+2rsf(1−f)

then the MT only transiently invades but declines to extinction in the long run. The competitive exclusion between WT and MT infections is shown in Fig. 4C, where we plot how the outcome depends on *s*, *f*, and *c*, given *r* = 0.5. Incorporating fitness parameters into the duration of infection gives similar results (results not shown). Furthermore, we show these results are robust to the addition of seasonality (Appendix 3).

In summary, the results of the epidemiological models show that the initial rate of invasion of the escape variant is similar to that in the population genetics model described by equation 2. However, at later time points, compensatory immunity reduces the rate of invasion. If the extent of CD8 T cell immunity to the escape variant is sufficiently high, the outcome may even be reversed if the overall selective advantage does not surmount the fitness cost.

## DISCUSSION

We considered the question of why CD8 T cell epitopes of human IAV are conserved. The answer to this question is relevant to the development of universal T cell-inducing vaccines against influenza and whether the virus is likely to evolve to escape the vaccine-induced immunity. Despite the wealth of empirical data showing conservation of CD8 T cell epitopes, the evolutionary mechanisms responsible for this conservation are not well understood. One possibility that has been widely considered is that the nucleoprotein and matrix-1 protein, which harbor the bulk of CD8 T cell epitopes, are under strong constraints (12, 13). In this view, mutations in CD8 T cell epitopes would have a high fitness cost or be relatively inaccessible due to epistatic changes needed for escape variants to have high fitness. In this study, we proposed two other mechanisms that could impact the fitness of viruses that escape CD8 T cell recognition. The first one is that escape from CD8 T cell responses against a single epitope provides only a relatively small selective advantage to the escape variant. The second one is that polymorphism in the MHC-I genes restricts this small advantage to only a small fraction of individuals in the population; individuals with other alleles would not present this epitope but present other epitopes. We show that even if there is a minimal fitness cost to having a mutation in a CD8 T cell epitope, the latter two factors will result in a very low rate of invasion of the CD8 T cell escape variant. We note that in the preceding models and discussion, we consider a scenario where invasion of a CD8 T cell escape variant is entirely driven by CD8 T cell selection. In reality, selection due to CD8 T cell immunity occurs in the context of a much stronger selection imposed by antibodies. The latter results in periodic replacement of virus strains over the course of a few seasons with drifted virus strains with novel antigenicity. We consider the consequences of this in more detail below.

The conclusion of this study seems to contradict the rapid invasion of the mutation at amino acid residue 384 of the nucleoprotein. This mutation alters the NP_383–391_ epitope presented by HLA-B*27:05 and the NP_380–388_ epitope presented by HLA-B*08:01. The wild-type sequence has an arginine (R), which forms an anchor residue at this site, and all 16 viruses isolated and sequenced during the 1992–1993 epidemic season had the wild-type sequence (23). An arginine-to-glycine mutation at this site (R384G) abrogates the MHC-I binding and prevents antigen presentation, and this mutation rapidly swept through the population in the 1993–1994 epidemic season, where all 56 virus isolates had G at this residue (23). Gog et al. (24) suggested that the rapid fixation of the R384G mutation was due to a combination of a longer duration of infection that slows its decline compared to that of the wild type over the summer and stochastic events. We propose an alternative hypothesis: the selective advantage of the R384G mutation is not required, and hitchhiking of a randomly generated mutant would be sufficient to explain the data. The rapid invasion of R384G temporally matches the transition from BE92 to WU95 antigenic clusters (25), suggesting that it hitchhiked with the antigenically drifted WU95 strain. Additionally, during 2002 to 2005, the invasion of the valine-to-isoleucine (V425I) mutant of the NP_418–426_ epitope was temporally associated with the transition from FU02 to CA04 (26). The rapid invasion pattern has also been observed on NP_251–259_ (invasion of S259L) and NP_103–111_ (alternating invasions of 103 K and 103R), all being consistent with the hitchhiking hypothesis.

Two recent analyses suggested that CD8 T cell epitopes of IAV circulating in the human population are under selection (27, 28). In particular, the number of CD8 T cell epitopes in circulating strains of influenza has gradually declined over a timescale of decades (28). For example, the H3N2 subtype had 84 experimentally confirmed epitopes per virus in 1968, and this number declined to 64 in 2015. A decline in the number of epitopes could be partially due to the bias in identification of epitopes, as new epitopes may not be identified and included. The observed decline rather than drift in the number of confirmed epitopes may arise because of a slight selective advantage of the escape variants over the wild type. This study suggests that the gradual escape of the virus from a CD8 T cell vaccine is possible.

We have intentionally used simple models, because the empirical data do not include accurate measurements of many of the key parameters that govern the generation and spread of virus escape variants. Under these circumstances, the results of simpler models are typically more robust than those of complex models (29). Our models can be thought of as simplified representations of the dynamics in the tropical regions, where the virus pool is maintained by continuous transmission. Antigenic changes arising in the tropical regions have been suggested to drive the seasonal epidemics in the Northern and Southern Hemispheres (30).

This study identifies the importance of measuring parameters such as the fitness of the wild-type and escape variants in hosts who have been previously infected with the wild type and both wild-type and escape variants. Aspects that might be included in more refined models are the waning of CD8 T cell immunity over time, particularly due to the loss of resident memory cells from the respiratory tract (31, 32), and the effect of stochasticity.

There are several differences in the ability of the influenza virus to evolve in response to antibody and CD8 T cell immunity. First, antibody immunity can generate sterilizing immunity (prevent infection with a matched virus strain) and, thus, generates substantial selection for antibody escape variants. In contrast, CD8 T cell immunity to influenza does not prevent infection and, thus, generates less selective advantage to a CD8 T cell escape variant. Second, an antibody escape variant will gain a selective advantage in the majority of individuals with antibodies to the wild-type strain, while a CD8 T cell escape variant will have a selective advantage only in individuals of a particular MHC-I genotype. Both of these factors contribute to antibody rather than CD8 T cell immunity driving antigenic drift in influenza.

Vaccination strategies that boost the CD8 T cell response may contribute to the development of broadly protective influenza vaccines. In this paper, we focused on whether these vaccine strategies will rapidly select for virus escape variants at CD8 T cell epitopes, compromising the effectiveness of the vaccine. We show that this is unlikely to be the case. Although it is generally viewed as a potential limitation that these vaccines may not completely prevent infection, this fact, together with MHC polymorphism, greatly reduces the selection pressure on the virus. Consequently, it may take a much longer duration for the virus to evolve and escape vaccine-induced CD8 T cell immunity.

## MATERIALS AND METHODS

### Population genetics model.

The analytical solution is found by rearranging equation 1 into

$$\frac{q_{t}}{1-q_{t}}=K\left(\frac{q_{t-1}}{1-q_{t-1}}\right)=K^{t}\left(\frac{q_{0}}{1-q_{0}}\right)$$

We then express *t*, the number of generations required for the MT to reach *q_t_*, as

$$t=\frac{1}{\text{log}\;K}\left[\text{log}\left(\frac{q_{t}}{1-q_{t}}\right)-\text{log}\left(\frac{q_{0}}{1-q_{0}}\right)\right]$$

The escape variant can invade when *K* > 1, i.e., *sf*^2^ + 2*rsf*(1 – *f*) > *m*.

### Map of CD8 T cell epitope on influenza nucleoprotein.

The epitope data set was retrieved from the Immune Epitope Database (www.iedb.org). We searched for an MHC class I-restricted linear epitope of influenza A virus (identifier 11320; FLUAV) in humans with at least one positive T cell assay. We retrieved 1,220 records from the IEDB, of which 514 were derived from NP. After excluding the records longer than 12 amino acid residues or with no HLA allele information available, records with the same amino acid sequence, records with different sequences but at the same location of NP and presented by the same HLA allele, or records nested under a longer record were combined into one unique epitope. In total, 64 unique epitopes were identified. Escape mutations were identified from the literature (9, 11).

An HLA allele data set reported by the NMDP was retrieved from The Allele Frequency Net Database (www.allelefrequencies.net). We included all alleles that have been reported to present at least one epitope in the epitope data set and calculated the average frequency weighted by sample sizes. In addition, since the alleles in one HLA supertype prefer amino acids with similar chemical properties at certain residues of the epitopes, we grouped the HLA alleles based on the classification proposed by Sidney et al. (33).

### Epidemiological model.

The ordinary differential equations corresponding to Fig. 3B are presented below.

$$\frac{\text{d}S_{W}^{j}}{\text{d}t}=\omega R_{W}^{j}-S_{W}^{j}\sum_{j=1}^{3}\left(\beta_{W}^{j}W_{W}^{j}+\mu_{W}^{j}M_{W}^{j}+\beta_{WM}^{j}W_{WM}^{j}+\mu_{WM}^{j}M_{WM}^{j}\right)$$

$$\frac{\text{d}S_{WM}^{j}}{\text{d}t}=\omega R_{WM}^{j}-S_{WM}^{j}\overset{3}{\underset{j=1}{\sum }}\left(\beta_{W}^{j}W_{W}^{j}+\mu_{W}^{j}M_{W}^{j}+\beta_{WM}^{j}W_{WM}^{j}+\mu_{WM}^{j}M_{WM}^{j}\right)$$

$$\frac{\text{d}W_{W}^{j}}{\text{d}t}=S_{W}^{j}\overset{3}{\underset{j=1}{\sum }}(\beta_{W}^{j}W_{W}^{j}+\beta_{WM}^{j}W_{WM}^{j})-\gamma W_{W}^{j}$$

$$\frac{\text{d}M_{W}^{j}}{\text{d}t}=S_{W}^{j}\overset{3}{\underset{j=1}{\sum }}\left(\mu_{W}^{j}M_{W}^{j}+\mu_{WM}^{j}M_{WM}^{j}\right)-\gamma M_{W}^{j}$$

$$\frac{\text{d}W_{WM}^{j}}{\text{d}t}=S_{WM}^{j}\overset{3}{\underset{j=1}{\sum }}\left(\beta_{W}^{j}W_{W}^{j}+\beta_{WM}^{j}W_{WM}^{j}\right)-\gamma W_{WM}^{j}$$

$$\frac{\text{d}M_{WM}^{j}}{\text{d}t}=S_{WM}^{j}\overset{3}{\underset{j=1}{\sum }}\left(\mu_{W}^{j}M_{W}^{j}+\mu_{WM}^{j}M_{WM}^{j}\right)-\gamma M_{WM}^{j}$$

$$\frac{\text{d}R_{W}^{j}}{\text{d}t}=\gamma W_{W}^{j}-\omega R_{W}^{j}$$

$$\frac{\text{d}R_{WM}^{j}}{\text{d}t}=\gamma (M_{W}^{j}+W_{WM}^{j}+M_{WM}^{j})-\omega R_{WM}^{j}$$

where *j* of 1, 2, and 3 denotes the genotype of *HH*, *Hh*, and *hh*, respectively. We started simulations from the equilibrium of WT infection, i.e.,

$$*S_{W}^{j}=\frac{\gamma }{\beta }\cdot \text{Freq}\left(j\right)$$

$$*I_{W}^{j}=\frac{\omega }{\omega + \gamma }\left(1-\frac{\gamma }{\beta }\right)\cdot \text{Freq}\left(j\right)$$

$$*R_{W}^{j}=\frac{\gamma }{\omega + \gamma }\left(1-\frac{\gamma }{\beta }\right)\cdot \text{Freq}\left(j\right)$$

where $\beta =\beta_{\text{W}}^{1}=\beta_{\text{W}}^{2}=\beta_{\text{W}}^{3}$ and Freq(*j*) is determined by *f* under Hardy-Weinberg equilibrium. Values of parameters are listed in Table 2; transmission parameters are in Table 3.

**TABLE 2:** Model parameters

Parameter	Symbol	Value
Transmission rate of WT infected^*a*^ (day^−1^)	β	0.4
Recovery rate (day^−1^)	γ	0.25
Drifting rate (day^−1^)	ω	0.0005

a $\beta =\beta_{W}^{j}=\beta_{WM}^{j}$; *j* = 1, 2, and 3. See Table 3 for the setup of transmission parameters in MT-infected subjects.

**TABLE 3:** Transmissibility according to genotypes and immune status

Compartment	Symbol	Value
$M_{W}^{1}$	$\mu_{W}^{1}$	$\beta \left(1-m\right)$
$M_{W}^{2}$	$\mu_{W}^{2}$	$\beta \left(1-m+rs\right)$
$M_{W}^{3}$	$\mu_{W}^{3}$	$\beta \left(1-m+s\right)$
$M_{WM}^{1}$	$\mu_{WM}^{1}$	$\beta \left(1-m\right)$
$M_{WM}^{2}$	$\mu_{WM}^{2}$	$\beta \left(1-m+rs\left(1-c\right)\right)$
$M_{WM}^{3}$	$\mu_{WM}^{3}$	$\beta \left(1-m+s\left(1-c\right)\right)$

## Figures and Tables

**FIG A1:**
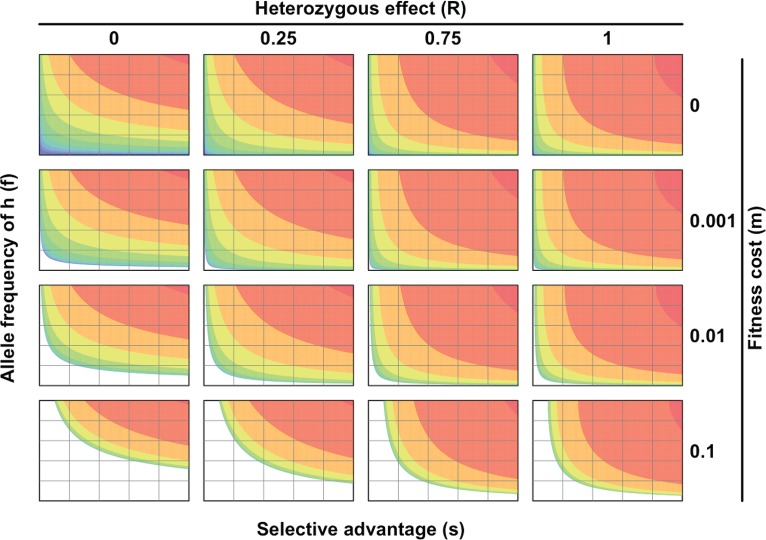
Number of generations for a CD8 T cell escape variant reaches 50% from 0.01% prevalence. The scale and color coding are the same as those in Fig. 1.

**FIG A2:**
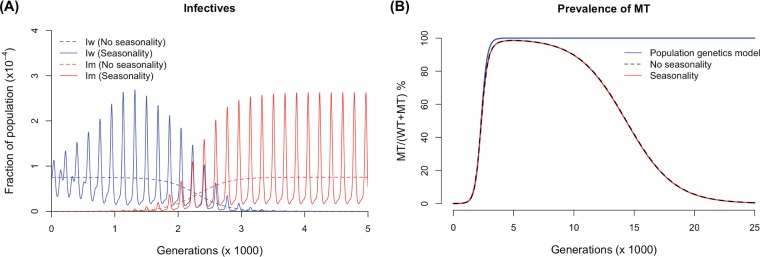
(A) Comparison between predictions of epidemiological model with (*A* = 0.02, *T* = 365 in equation A1) and without (*A* = 0) seasonality. Other parameters are kept the same as those in Fig. 4A. (B) Comparison between predicted MT prevalence from population genetics model, epidemiological with and without seasonality.

## APPENDIX

### Population genetics model.

Figure A1 shows the number of generations for a CD8 T cell escape variant (MT) to reach 50% from 0.01% prevalence with different fitness costs (*m* = 0, 0.001, 0.01, and 0.1) and dominance coefficients (*r* = 0, 0.25, 0.75, and 1). As *m* increases, the MT requires higher *s* and/or *f* to overcome the deleterious effect. The increase in *r* changes the shape of the contours, since it raises the selective advantage of MT in the heterozygotes. As a result, the threshold for MT invasion when *s* is large is lower than that when *r* is small.

### Quantifying selection pressure on virus.

We define three types of immunity effectiveness (IE): IE*_S_* is the probability that an individual who has influenza-specific CD8 T cells does not get infected upon contact with an infectious individual, IE*_P_* is the probability that an infected individual who has influenza-specific CD8 T cells does not develop symptoms, and IE*_I_* is the probability that an infected individual who has influenza-specific CD8 T cells does not spread the virus.

The selection pressure on virus S is formulated by 1 − S = (1 − IE*_S_*) (1 − IE*_I_*). The viral fitness after selection, 1 − S, is the probability of transmission within the host population who have influenza-specific CD8 T cells, and it can be expressed as the probability that an immune individual gets infected (1 − IE*_S_*) and spreads the virus (1 − IE*_I_*).

### Effect of seasonality.

Seasonality is incorporated into transmissibility by

A1 βij(t)=βij[1+A sin(2πtT)]

where *i* and *j* indicate the host's immune status and genotype, respectively, 0 ≤ *A* ≤ 1 is the amplitude of oscillation, and *T* is the period (34). Simulation of model with *A* = 0.02 and *T* = 365 (annual outbreaks with β ranging from 0.392 to 0.408) was compared to the one with *A* = 0 (no seasonality). Our results show that the mean oscillation is about the same as that seen in the model with no seasonality (Fig. A2).
